# Surgical site infection surveillance in German hospitals: a national survey to determine the status quo of digitalization

**DOI:** 10.1186/s13756-023-01253-9

**Published:** 2023-05-19

**Authors:** Seven Johannes Sam Aghdassi, Hengameh Goodarzi, Alexander Gropmann, Jörg Clausmeyer, Christine Geffers, Brar Piening, Petra Gastmeier, Michael Behnke

**Affiliations:** 1 Charité – Universitätsmedizin Berlin, corporate member of Freie Universität Berlin and Humboldt-Universität zu Berlin, Institute of Hygiene and Environmental Medicine, Hindenburgdamm 27, 12203 Berlin, Germany; 2 National Reference Center for Surveillance of Nosocomial Infections, Hindenburgdamm 27, 12203 Berlin, Germany; 3 Berlin Institute of Health at Charité – Universitätsmedizin Berlin, BIH Biomedical Innovation Academy, BIH Charité Digital Clinician Scientist Program, Anna-Louisa-Karsch-Straße 2, 10178 Berlin, Germany

**Keywords:** Automation, Digitalization, Surveillance, Surgical site infection, Healthcare-associated infection, Digital infection control

## Abstract

**Background:**

Surveillance of surgical site infections (SSI) relies on access to data from various sources. Insights into the practices of German hospitals conducting SSI surveillance and their information technology (IT) infrastructures are scarce. The aim of this study was to evaluate current SSI surveillance practices in German hospitals with a focus on employed IT infrastructures.

**Methods:**

German surgical departments actively participating in the national SSI surveillance module “OP-KISS” were invited in August 2020 to participate in a questionnaire-based online survey. Depending on whether departments entered all data manually or used an existing feature to import denominator data into the national surveillance database, departments were separated into different groups. Selected survey questions differed between groups.

**Results:**

Of 1,346 invited departments, 821 participated in the survey (response rate: 61%). Local IT deficits (n = 236), incompatibility of import specifications and hospital information system (n = 153) and lack of technical expertise (n = 145) were cited as the most frequent reasons for not using the denominator data import feature. Conversely, reduction of workload (n = 160) was named as the main motivation to import data. Questions on data availability and accessibility in the electronic hospital information system (HIS) and options to export data from the HIS for the purpose of surveillance, yielded diverse results. Departments utilizing the import feature tended to be from larger hospitals with a higher level of care.

**Conclusions:**

The degree to which digital solutions were employed for SSI surveillance differed considerably between surgical departments in Germany. Improving availability and accessibility of information in HIS and meeting interoperability standards will be prerequisites for increasing the amount of data exported directly from HIS to national databases and laying the foundation for automated SSI surveillance on a broad scale.

**Supplementary Information:**

The online version contains supplementary material available at 10.1186/s13756-023-01253-9.

## Background

Surgical site infections (SSI) represent one of the most frequently occurring types of healthcare-associated infections (HAI) and entail a substantial burden of disease [1–3]. Surveillance of SSI has been demonstrated repeatedly to effectively prevent infections and reduce SSI rates [4, 5], and is therefore recommended as a preventive strategy by the World Health Organization [6, 7]. SSI surveillance in Germany has a longstanding tradition and is organized in the module “OP-KISS” of the national surveillance network “KISS” (German: *Krankenhaus-Infektions-Surveillance-System*) [8]. The German National Reference Center for Surveillance of Nosocomial Infections (NRC) organizes and coordinates surveillance activities in KISS. Over 1000 surgical departments regularly participate in OP-KISS [9, 10]. Participating departments are located primarily in Germany, and in smaller numbers in Austria and Switzerland. Participation in OP-KISS is voluntary and data is primarily intended for internal quality assessment. Additionally, data is transmitted by OP-KISS participants to the NRC, enabling the NRC to calculate aggregated reference data. OP-KISS is based on so-called “indicator procedures” that each comprise various procedure codes. Participants in OP-KISS can freely choose for which indicator procedures they perform SSI surveillance. As specified in the OP-KISS methodology, participating departments collect data for eligible procedures and observe patients for SSI occurrence for a defined period, 30 or 90 days depending on the type of indicator procedure. Surveillance ends prematurely in case of reoperation or death [11]. Data collection and interpretation as well as data transfer to the NRC are performed by staff at the local hospital. For data transfer to the NRC, participating departments must use a specific surveillance web portal “webKess” (https://webkess.charite.de/), into which data can either be entered manually or imported. Data import generally pertains to denominator data, although import of numerator data (i.e. SSI) is possible.

Conventionally, SSI surveillance relies on a manual process to identify eligible procedures and subsequently observe operated patients concerning SSI occurrence. Accordingly, conventional SSI surveillance frequently represents a laborious and time-consuming process, usually resulting in the pragmatic but restrictive decision to observe only selected types of surgeries [12, 13]. Automation of certain work steps may offer an important enhancement to HAI surveillance in general and SSI surveillance in particular, and a means to reduce the required workload [14–17]. Unlike several other countries [17], no large-scale automated SSI surveillance systems exist yet in Germany. This may be due to digitalization deficits in German hospitals and heterogeneity regarding the employed hospital information systems (HIS). The abovementioned option to import denominator data into webKess, however, represents a first step in the direction of utilizing information technology (IT) solutions for surveillance, and possibly automated surveillance. Neither the degree to which IT solutions are currently harnessed to conduct surveillance, nor perceived challenges and initiatives for doing so, have been described for German hospitals, rendering it difficult to assess the full potential of automated SSI surveillance in Germany.

To better understand the current state of data collection methods and use of digital infrastructures for SSI surveillance in Germany, the NRC conducted a survey among OP-KISS participants.

## Methods

In 2020, the NRC created a survey to be sent out to surgical departments actively participating in OP-KISS. Active participation was defined as having transferred SSI surveillance data for 2018 or 2019 to the NRC. Departments invited to participate were located in Germany, Austria or Switzerland. Actively participating departments were divided into three groups, based on whether or not they had used the webKess import function in the previous two years. “Group A” was defined as departments that had entered data for the years 2018 and 2019 only manually. “Group B” consisted of departments that had imported denominator data for 2018, but not 2019. Departments that had imported denominator data for 2019 constituted “group C”, irrespective of their mode of data entry in 2018. The survey followed the same structure for all three groups and questions were mostly identical. Only where considered necessary by the investigators, specific questions differed between groups. In all cases, the survey comprised of ten questions, with additional sub-questions that had to be answered only if certain answers to the original ten questions were selected by the respondents. Thematically, the survey can be divided into three topics: general IT and HIS aspects; the webKess import feature and reasons for using or not using it; and strategies employed in the practice of SSI surveillance.

The survey was conducted online using Limesurvey (https://www.limesurvey.org/). The survey language was German. An English translation of the survey documents can be found in the online supplement (Additional File 1). An invitation to participate in the survey was sent to the main contact person for every included department on August 4, 2020. Data entry was possible until September 30, 2020. A reminder was sent on September 1, 2020. Participation in the survey was voluntary. Multiple departments per hospital could participate in the survey, but participation was only possible once per individual department. The survey had to be fully completed in one session. Once the survey was finished, all entered data were automatically sent to the NRC. After that, participants could no longer modify responses. In cases where participants realized afterwards that mistakes had been made, corrections could be requested by contacting the study team.

After reception of the responses, the NRC evaluated the data. For the purpose of this analysis, questions of particular value to describe the current state of data collection methods and employed digital infrastructures in the context of SSI surveillance in Germany were selected, and datasets from Austrian or Swiss departments were excluded. Included survey datasets were matched with data on structural characteristics and number of surgeries under surveillance from the OP-KISS database. As will be demonstrated in the results section below, only few datasets from group B were received. In response to this, the study team decided to combine groups B and C when presenting most survey results. The decision to combine group B with group C, instead of group A, was made based on the consideration that departments in both group B and group C had used the import feature for at least one of the two years. A separate presentation of results from groups B and C will be given only when necessary due to differing questions between survey versions.

## Results

A total of 1,346 German surgical departments from 707 hospitals received an invitation to participate in the survey. Altogether, 821 surgical departments (department response rate: 61%) from 469 hospitals (hospital response rate: 66%) conducted the survey. Stratified by the defined groups, of 1021 invited group A departments, 605 participated in the survey (response rate: 59%), of 35 invited group B departments, 23 participated (response rate: 66%), and of 290 invited group C departments, 193 conducted the survey (response rate: 67%).

Participating departments in group A were from hospitals with a considerably lower median number of beds than departments in groups B&C (327 vs. 490). Whereas the percentage of departments that were located in public hospitals was comparable between survey groups (28% group A vs. 32% groups B&C), differences were noted concerning the percentage of departments from tertiary or maximum care hospitals (35% in group A vs. 47% in groups B&C). The median number of procedures per department transmitted to the NRC for the years 2018 and 2019 was lower in group A (291) than groups B&C (315). Further structural characteristics of participating departments are illustrated in Table 1.

**Table 1:** Structural characteristics at the hospital level and number of procedures transmitted to the national reference center of 605 German surgical departments in group A and 216 German surgical departments in groups B&C that participated in the survey

Variable	Group A Number (percentage) or Median (interquartile range)	Groups B&C Number (percentage) or Median (interquartile range)
Number of hospital beds	327 (204, 563)	490 (305, 686)
Departments in tertiary or maximum care hospitals	211 (34.9)	101 (46.8)
Departments in non-tertiary, non-maximum care* hospitals	394 (65.1)	115 (53.2)
Departments in public hospitals	171 (28.3)	69 (31.9)
Departments in non-public^#^ hospitals	434 (71.7)	147 (68.1)
Number of procedures under surveillance in 2018 and 2019^§^	291 (156, 532)	315 (163, 626)

^*^Contains primary care, secondary care, specialized care and unspecified. ^#^Contains private for profit, private non-profit, ecclesiastical, other and unspecified. ^§^ Refers to all transmitted procedures, incl. ones marked as “during surveillance pause” or “not valid for reference data”

### General IT and HIS aspects

The majority (53% [434 of 821]) of survey participants reported utilizing an infection prevention and control (IPC) software from an external provider that can assist the extraction of data relevant to HAI surveillance from the HIS. Differentiated by group, 46% (279 of 605) of departments in group A, and 72% (155 of 216) of departments in groups B&C reported employing external IPC software. While 50% (303 of 605) of departments in group A reported receiving support for their surveillance activities from the hospital IT team, this number was 75% (161 of 216) for groups B&C. Of the 302 departments in group A that reported not already receiving IT support for their surveillance activities, 96 (32%) saw the prospect for IT support in the future, in groups B&C the same assessment was made by 27% (15 of 55) of departments.

Table 2 documents the availability of important variables for SSI surveillance in the HIS. Small differences concerning the availability of data between groups A and B&C were observed, for instance regarding the availability of the wound contamination class (67% group A vs. 77% groups B&C).

**Table 2:** Availability of variables for surgical site infection surveillance in the hospital information system. Responses from 605 German surgical departments in group A and 216 German surgical departments in groups B&C

Variable	Available in HIS—Group A Number (percentage)	Available in HIS—Groups B&C Number (percentage)
Type of surgery (procedure code)	601 (99.3)	216 (100)
Date of surgery	600 (99.2)	215 (99.5)
Age (year of birth)	601 (99.3)	216 (100)
Sex	598 (98.8)	216 (100)
Date of hospital admission	594 (98.2)	209 (96.8)
Date of hospital discharge	595 (98.3)	208 (96.3)
ASA score	536 (88.6)	203 (94.0)
Wound contamination class	403 (66.6)	166 (76.9)
Duration of surgery	596 (98.5)	210 (97.2)
Endoscopic (yes or no)*	500 (82.6)	172 (79.6)
Urgent^#^ procedure (yes or no)*	449 (74.2)	159 (73.6)
Revision surgery (yes or no)*	432 (71.4)	152 (70.4)
Implant (yes or no)*	483 (79.8)	164 (75.9)
Surgical site infection data	348 (57.5)	122 (56.5)
Premature end of surveillance (due to reoperation or death)	401 (66.3)	143 (66.2)

^*^Collected only for selected types of indicator procedures. ^#^According to the OP-KISS methodology, urgent procedures are surgeries that were not planned 24 h or longer in advance. Abbreviations: ASA: American Society of Anesthesiologists; HIS: hospital information system

A survey question focusing on the electronic availability of microbiological findings important for SSI surveillance (e.g. wound swabs) yielded congruous results between groups. In group A, 90% (542 of 605) of departments reported that microbiological findings were available electronically. In groups B&C, responses were similar (94% [203 of 216]). Departments that stated that microbiological results were available electronically, were asked to further specify whether they were available in a structured and machine-readable format (e.g. FHIR®, CSV, HL7 v2.x). Here, 34% (182 of 542) of departments in group A stated that this was the case. In groups B&C, the percentage was considerably higher (60% [121 of 203]).

### webKess import function

**Fig. 1:**
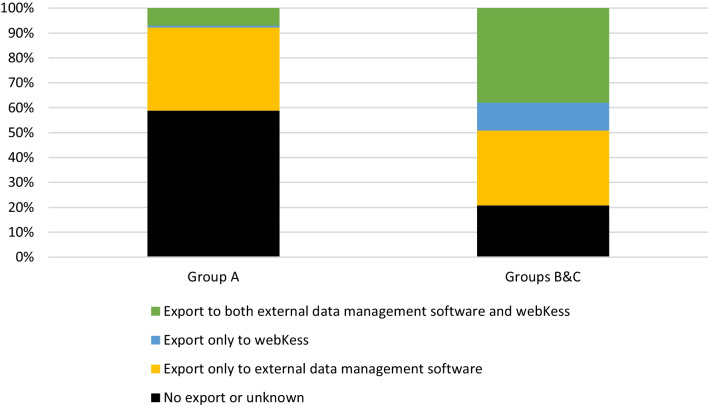
Feasibility of data export from the hospital information system. Responses from 605 German surgical departments in group A and 216 German surgical departments in groups B&C

When asked whether data from the HIS could be exported to an external data management software (e.g. Microsoft® Excel®) and/or directly to webKess, 41% (249 of 605) of departments in group A replied that this was possible, while 79% (171 of 216) of departments in groups B&C did so. Further information, including a distinction whether export was possible to both an external data management software and webKess, or to only one of the two, is provided in Fig. 1. The figure reveals that particularly the possibility to export directly from the HIS to webKess is lower in group A than groups B&C.

Departments that reported that data export from the HIS was possible, were additionally asked to specify the parameters with significance to SSI surveillance that could be exported. The responses are summarized in Table 3. In general, availability of variables for export was higher in groups B&C than in group A.

**Table 3:** Parameters exportable from the hospital information system

Parameter	Exportable from HIS—Group A Number (percentage)	Exportable from HIS—Groups B&C Number (percentage)
Type of surgery (procedure code)	241 (96.8)	169 (98.8)
Date of surgery	238 (95.6)	169 (98.8)
Age (year of birth)	236 (94.8)	169 (98.8)
Sex	223 (89.6)	165 (96.5)
Date of hospital admission	206 (82.7)	151 (88.3)
Date of hospital discharge	209 (83.9)	142 (83.0)
ASA score	174 (69.9)	155 (90.6)
Wound contamination class	137 (55.0)	141 (82.5)
Duration of surgery	205 (82.3)	163 (95.3)
Endoscopic (yes or no)	150 (60.2)	131 (76.6)
Urgent^#^ procedure (yes or no)	121 (48.6)	104 (60.8)
Revision surgery (yes or no)	97 (39.0)	100 (58.5)
Implant (yes or no)	122 (49.0)	125 (73.1)
Surgical site infection data	81 (32.5)	72 (42.1)
Premature end of surveillance (due to reoperation or death)	74 (29.7)	79 (46.2)

Responses from 249 German surgical departments in group A and 171 German surgical departments in groups B&C that reported that data export from the hospital information system was possible

^#^According to the OP-KISS methodology, urgent procedures are surgeries that were not planned 24 h or longer in advance. Abbreviations: ASA: American Society of Anesthesiologists; HIS: hospital information system

In group A, 241 departments reported that they could export the type of surgery (i.e. the procedure code) from the HIS, in groups B&C, it was 169 departments respectively. These departments were requested to specify how the allocation from procedure code to the corresponding OP-KISS indicator procedure type was executed. Here, 46% (111 of 241) in group A, and 89% (150 of 169) in groups B&C reported that this was performed automatically, either as a feature of the export from the HIS, or by directly importing the procedure code into webKess. Conversely, 49% (118 of 241) of departments in group A and 9% (16 of 169) in groups B&C reported that this was done manually by staff. The remaining departments either specified another method or did not provide a response.

To learn more about potential hurdles of a direct export of HIS data to webKess, participants that reported they could export HIS data to an external data management software but not to webKess directly, were asked which parameters required manual editing before import into webKess. The responses are summarized in Table 4 and demonstrate that manual editing is necessary more frequently in group A than groups B&C.

**Table 4:** Exportable parameters from the hospital information system requiring manual editing before import into webKess

Parameter	Requiring manual editing—Group A Number (percentage)	Requiring manual editing—Groups B&C Number (percentage)
Type of surgery (procedure code)	106 (52.5)	5 (7.7)
Date of surgery	63 (31.2)	8 (12.3)
Age (year of birth)	65 (32.2)	8 (12.3)
Sex	73 (36.1)	8 (12.3)
Date of hospital admission	76 (37.6)	3 (4.6)
Date of hospital discharge	76 (37.6)	7 (10.8)
ASA score	115 (56.9)	20 (30.8)
Wound contamination class	134 (66.3)	30 (46.2)
Duration of surgery	85 (42.1)	10 (15.4)
Endoscopic (yes or no)	102 (50.5)	18 (27.7)
Urgent^#^ procedure (yes or no)	112 (55.4)	29 (44.6)
Revision surgery (yes or no)	123 (60.9)	25 (38.5)
Implant (yes or no)	117 (57.9)	29 (44.6)
Surgical site infection data	159 (78.7)	55 (84.6)
Premature end of surveillance (due to reoperation or death)	154 (76.2)	39 (60)

Responses from 202 German surgical departments in group A and 65 German surgical departments in groups B&C that reported export from the hospital information system was possible only to an external data documentation software, but not directly to webKess

^#^According to the OP-KISS methodology, urgent procedures are surgeries that were not planned 24 h or longer in advance. Abbreviations: ASA: American Society of Anesthesiologists; HIS: hospital information system

Depending on the group that departments were allocated to, survey questions exploring recent use or non-use of the webKess import feature differed. Departments in group A had to state, why they had not previously used the import function. The primary reasons provided were local IT deficits (n = 236), incompatibility of webKess import specifications and HIS (n = 153), and lack of technical expertise (n = 145). Departments in group B had to state, why they had discontinued using the import function. Lack of technical expertise (n = 4) was the most frequently provided answer, with most other answers being provided as free text often citing local structural and process changes. Departments in group C were asked to state the reasons for utilizing the import function, with reduction of workload (n = 160) most commonly reported. Furthermore, departments in group C were asked whether they used the import function, not only for denominator data but also for numerator data (i.e. data on SSI), with 54% (104 of 193) reportedly doing so.

### Practice of SSI surveillance

When asked to describe the process of SSI surveillance, responses between group A and groups B&C were largely consistent, with review of microbiological findings (95% [573 of 605] in group A, 92% [199 of 216] in groups B&C) and actively inquiring updates of treating staff (62% [378 of 605] in group A, 72% [155 of 216] in groups B&C) being the most common regularly (i.e. “frequently”, “very frequently” or “always” selected as response) performed surveillance strategies in both groups. Moreover, participants were asked whether they regularly continued surveillance after patients were discharged from the hospital (so-called “post-discharge surveillance”). Post-discharge surveillance was reportedly performed systematically by 42% (253 of 605) of departments in group A, and 32% (69 of 216) of departments in groups B&C.

## Discussion

To the best of our knowledge, this survey provides the first detailed description of IT infrastructures used by hospitals for conducting SSI surveillance within a large national surveillance network. To inform a better understanding of the international situation regarding this matter, and to strengthen international cooperation in the field of SSI surveillance, we wish to encourage other countries and surveillance networks to conduct similar surveys.

As was expected, analysis of the survey results revealed heterogeneity concerning availability and utilization of IT options in the practice of SSI surveillance among German surgical departments. Although not an outcome parameter of the survey itself, this becomes apparent already when comparing the number of departments per group that were invited to participate in the survey. Group A, which was defined by manual data entry into webKess, contained more than three times as many departments than groups B&C, which had imported denominator data into webKess in at least one of the two considered years, illustrating that SSI surveillance is still largely a manual process in Germany. Given that automated HAI surveillance and even automated identification of eligible operations (i.e. denominator data) can be a means to save much needed resources [15, 17, 18], the observed distribution of departments into the respective groups, documents a high unused potential to save IPC resources in Germany. This interpretation is supported, when considering that reduction of workload was stated as the primary motivation for using the webKess import feature.

Departments utilizing the import feature were found to be from larger hospitals and from hospitals with a higher level of care than departments relying solely on manual data entry. This finding seems to corroborate the somewhat intuitive assumption that tertiary care hospitals and larger hospitals in general have more technical options at their disposal. Interestingly, differences between groups with regards to public versus private hospital ownership were small. The fact that the number of procedures transmitted to the NRC for the years 2018 and 2019 was higher in groups B&C suggests that time conventionally spend on manual entry of denominator data, can be reallocated to perform SSI surveillance for a higher number of procedures. This interpretation is in alignment with studies concluding that automating certain aspects of SSI surveillance offers potentials to increase the number of observed procedures [14].

Our survey offered valuable insights into the underlying reasons for the high number of departments still relying solely on manual data entry. Only half of departments in group A reported receiving IT support for conducting surveillance, whereas in groups B&C IT support was available considerably more often (circa 75%). Moreover, the fact that only around one third of departments not already receiving IT support, were hopeful to receive support in the future, revealed significant shortcomings concerning IT support. This interpretation is reinforced when considering the stated reasons, why the webKess import feature had not been used. IT deficits, technical incompatibilities and lack of technical expertise were seen as the main barriers to data import. Our survey therefore highlights the importance of prioritizing interprofessional cooperation and support from dedicated IT teams, when setting up structures for HAI surveillance. The significance of tailoring local systems to perform surveillance functions has been discussed in previous publications [19, 20]. Evidently, local IT support represents a prerequisite for this process.

Our survey uncovered additional factors forcing IPC staff to perform surveillance manually, beyond lack of IT support and expertise. While the general availability of variables for SSI surveillance in HIS was comparable between the different survey groups, pronounced differences were noted concerning the option to export data from HIS. Fewer than half of departments in group A reported that exporting surveillance data from HIS was possible, which was substantially lower than for groups B&C. It is important however to be mindful, that documented deficits could be to a certain extent be overestimated by the fact that some respondents, due to lack of adequate local IT support, might have been unaware of data export options that actually existed but were merely not utilized. Both interpretations however, the lack of specific IT features or the missed opportunity of using existing features, highlight the importance of considering surveillance use cases when designing or respectively selecting HIS, and the significance of ensuring interoperability between systems linked to the process of HAI surveillance [21].

Our survey provides further insights into this matter by detailing for individual surveillance parameters, whether data export from HIS was possible, and whether manual editing of data before webKess import was necessary. It is particularly critical that important procedure-related variables, such as wound contamination class, surgical access route (endoscopic vs. open), duration of surgery and American Society of Anesthesiologists (ASA) score, were frequently reported to be not exportable from HIS, or requiring manual editing if they were. This finding is to a certain extent surprising, since wound contamination class, ASA score and duration of surgery are used for risk stratification in OP-KISS [22]. Similarly, around half of departments in group A reported that the allocation from procedure code to the corresponding OP-KISS indicator procedure was performed manually by staff. According to the OP-KISS methodology, the allocation to the correct indicator procedure is a prerequisite to collect any useful surveillance data at all [11]. Therefore, the need for a manual process to assign an operation to the appropriate indicator procedure must be viewed as a clear potential for improvement.

The results and parameters discussed thus far predominately pertained to denominator data. However, when trying to explore potentials to automate SSI case finding, other variables should be considered as well. Various variables have been identified to yield particular value for automated SSI surveillance, for instance, microbiological findings, hospital admissions, (revision) surgeries, and antimicrobial prescriptions [14]. To gain insights into this aspect, a question regarding the availability of microbiological findings was included in the survey. In both group A and groups B&C, electronic availability of this information was widespread. While this can be viewed as a promising potential for automation, differences between the groups concerning the format and machine-readability of microbiological results, call for a more nuanced interpretation. Groups B&C were found to have microbiological results available in a structured and machine-readable format decidedly more often than group A (60% vs. 34%). However, even in groups B&C more than a third of departments reported that microbiological results were not available in a structured and machine-readable format, which illustrates that for departments from both groups harnessing microbiological data for automated surveillance might be challenging. This once again stresses the crucial role of ensuring data standardization and meeting interoperability standards in the context of HAI surveillance [21].

In a separate section of the survey, the practice of SSI surveillance by participating departments was investigated. Irrespective of the survey group, review of microbiological results and active information gathering from treating staff, were named as commonly employed surveillance strategies. As delineated above, interpretation of microbiological results entails a high potential for automation. Provided a consistent way of documenting the clinical course of patients after surgery, information gathering from treating staff could be assisted by algorithms searching for key terms in the patient file, thus also representing a strategy that could be partly automated. This interpretation is reinforced by the fact that over half of departments in group C reported having used the webKess import function also for numerator data. Similarly, the practice of post-discharge surveillance should be considered in future automated surveillance strategies, given that between one third and one half of departments reported performing it systematically. The crucial role of adequate post-discharge surveillance for detecting a substantial portion of SSI has been described in various publications [23–25]. The Hospital-Acquired Infections Database (HAIBA) from Denmark represents a prime example of intersectoral data exchange for the purpose of continuing HAI surveillance after hospital discharge [26].

Several limitations have to acknowledged when interpreting the survey results. First, the survey was not distributed to a representative sample of surgical departments, but to all OP-KISS participants that met the inclusion criteria. Consequently, statements concerning the national situation have to be made with caution. Nevertheless, due to the large number of participating departments, careful generalizations to the national situation appear to be warranted. Second, the survey was based on voluntary participation. Accordingly, departments with a particular interest in the survey topic may be overrepresented, which could distort survey results towards an overestimation of the use of IT infrastructures for surveillance. Third, although all data was handled confidentially, some survey questions might have been perceived as potentially compromising, which could result in “wishful reporting”. However, given the long trust-building history of conducting surveys in the KISS network [27–29], we assess this risk to be rather low. Forth, certain questions, specifically when pertaining to technical aspects, might have been difficult to understand for some survey recipients, which were typically IPC professionals. To counteract this, survey participants were encouraged to seek assistance from other professional groups (e.g. IT team) whenever necessary. Nevertheless, responses indicating the non-availability of data or specific features, particularly pertaining to data export and import, might in some cases not accurately reflect the actual situation, but rather a lack of knowledge of the respondent. Last, if data was entered erroneously, participants could not perform corrections themselves, but had to contact the study team, requiring more effort than simply re-entering the questionnaire and changing a response. Thus, the analyzed dataset might have contained incorrect responses. However, to reduce this risk to a minimum, participants were advised to print out the survey on paper, and fill in answers before entering data into the online survey template.

## Conclusions

IT infrastructures play an important part in the practice of SSI surveillance in Germany. The degree, to which they are harnessed, however, varies considerably between surgical departments. Local IT deficits, technical difficulties and general lack of local IT support, were found to hinder the use of existing data import features. To increase the amount of data exported directly from local HIS to the national surveillance database, and therefore lay the foundation for automated SSI surveillance in Germany, hospitals should seek solutions to improve availability and accessibility of information in HIS, and ensure necessary data standardization as well as adherence to interoperability standards. The results of our survey strongly indicate that hospitals in Germany and their digital subsystems lag far behind contemporary standards.

## Supplementary Information

**Additional file 1**. Translated version of the OP-KISS survey on SSI surveillance and digitalization.

## Data Availability

Not applicable, because all data were surveillance-based data which were obtained in accordance with the German Protection against Infection Act.

## Abbreviations

ASA: American society of anesthesiologists
HAI: Healthcare-associated infection(s)
HAIBA: Hospital-acquired infections database
HIS: Hospital information system(s)
IPC: Infection prevention and control
IT: Information technology
KISS: Krankenhaus-infektions-surveillance-system
NRC: National reference center
SSI: Surgical site infection(s)
