# Supplementary material for: Surgical site infection surveillance in German hospitals: a national survey to determine the status quo of digitalization
Source: Antimicrob Resist Infect Control. 2023 May 19;12:49. doi: 10.1186/s13756-023-01253-9 (PMC10197484; doi:10.1186/s13756-023-01253-9)
Supplement: Supplementary file 1 — Additional file 1. Translated version of the OP-KISS survey on SSI surveillance and digitalization. [file 13756_2023_1253_MOESM1_ESM.docx]

**Surgical site infection surveillance in German hospitals: a national survey to determine the status quo of digitalization**

**Survey: Group A**

**1. Which hospital information system (HIS) do you use in your hospital?** *(Please select one answer)*

- SAP / i.s.h.med
- ORBIS
- NEXUS
- Cerner / Siemens Medico
- Unknown
- Other, [please specify:] ___________ *(free text)*

**2. Do you use an external infection prevention and control software?** *(Please select one answer)*

- Yes
- No
- Unknown

**2.1. Which one?** *(To be answered if answer to question 2 was “Yes”) (Please select one answer)*

- QS-MED (3M™)
- HyBASE® (epiNet AG)
- MetaIPSS (Cerner)
- MetaHMS (Cerner)
- MedReport (Dorner)
- ORBIS Infektionsmanagement (AGFA Healthcare)
- Other, [please specify:] ___________ *(free text)*

**3. Does your hospital provide IT support that assists you in your surveillance activities?** *(Please select one answer)*

- Yes
- No

**3.1. Does your hospital have IT support that could assist you in your surveillance activities?** *(To be answered if answer to question 3 was “No”) (Please select one answer)*

- Yes
- No
- Unknown

**3.1.1. Optional Question: Why do you not receive IT support for surveillance activities in your hospital?** *(To be answered if answer to question 3.1 was “No”) (Please provide an answer as free text)*

- ___________ *(free text)*

**4. Is the following information regarding operations available in your hospital information system (HIS)?** *(Please select all that apply)*

- Type of surgery (procedure code)
- Date of surgery
- Age (year of birth)
- Sex
- Date of hospital admission
- Date of hospital discharge
- ASA score
- Wound contamination class
- Duration of surgery
- Endoscopic (yes or no)
- Urgent procedure (yes or no)
- Revision surgery (yes or no)
- Implant (yes or no)
- Surgical site infection data
- Premature end of surveillance (due to reoperation or death)
- Unknown
- Other, [please specify:] ___________ *(free text)*

**5. Do you use an [external] data management software (e.g. Microsoft® Excel®) to conduct your surveillance?** *(Please select one answer)*

- Yes
- No
- Unknown

**5.1.** **Which one?** *(To be answered if answer to question 5 was “Yes”) (Please select one answer)*

- Microsoft® Excel®
- Other, [please specify:] ___________ *(free text)*

**6. Is it possible for you to export data from the hospital information system (HIS) (e.g. to Microsoft® Excel® or webKess)?** *(Please select one answer)*

- Yes, export to both [external] data management software (e.g. Microsoft® Excel®) and webKess
- Yes, export to [external] data management software (e.g. Microsoft® Excel®), but not to webKess
- Yes, export to webKess, but not to [external] data management software (e.g. Microsoft® Excel®)
- No export possible
- Unknown

**6.1. Which data can be exported?** *(To be answered if answer to question 6 was “Yes, …”) (Please select all that apply)*

- Type of surgery (procedure code)
- Date of surgery
- Age (year of birth)
- Sex
- Date of hospital admission
- Date of hospital discharge
- ASA score
- Wound contamination class
- Duration of surgery
- Endoscopic (yes or no)
- Urgent procedure (yes or no)
- Revision surgery (yes or no)
- Implant (yes or no)
- Surgical site infection data
- Premature end of surveillance (due to reoperation or death)
- Other, [please specify:] ___________ *(free text)*

**6.1.1. How is the allocation from surgical procedure code to KISS indicator procedure performed (e.g. procedure code 5-455.1 corresponds to KISS indicator procedure “COLO”)?** *(To be answered if “Type of surgery (procedure code)” was selected in question 6.1) (Please select one answer)*

- Automatically through the export from the hospital information system (HIS)
- Manually by [staff] (e.g. paper-based, via an [external] data management software (e.g. Microsoft® Excel®)
- Automatically [through] a direct import of the procedure code into webKess
- Unknown
- Other, [please specify:] ___________ *(free text)*

**6.2. Which [exportable parameters]/data must be manually edited before they can be entered or imported into webKess?** *(To be answered if answer to question 6 was “Yes, export to [external] data management software (e.g. Microsoft® Excel®), but not to webKess”) (Please select all that apply)*

- Allocation of procedure code to KISS indicator procedure (e.g. procedure code 5-455.1 corresponds to KISS indicator procedure “COLO”)
- Date of surgery
- Age (year of birth)
- Sex
- Date of hospital admission
- Date of hospital discharge
- ASA score
- Wound contamination class
- Duration of surgery
- Endoscopic (yes or no)
- Urgent procedure (yes or no)
- Revision surgery (yes or no)
- Implant (yes or no)
- Surgical site infection data
- Premature end of surveillance (due to reoperation or death)
- Unknown
- Other, [please specify:] ___________ *(free text)*

**7. Why has your department not previously used the webKess import functionality for [surveillance] data?** *(Please select all that apply)*

- Local IT deficits
- Lack of technical expertise
- [webKess] import specifications are incompatible with hospital information system (HIS) or other employed system
- Complexity of [webKess] import specifications
- Lack of necessity
- Lack of personnel
- Other, [please specify:] ___________ *(free text)*

**8. In which form are microbiological results (e.g. wound swab, blood culture) necessary for OP-KISS surveillance available?** *(Please select one answer)*

- Electronically and on paper
- [Only] electronically
- [Only] on paper
- Not available at all

**8.1a. Please specify, how microbiological results are available electronically.** *(To be answered if answer to question 8 was “Electronically and on paper” or “[Only] electronically”) (Please select one answer)*

- In the hospital information system (HIS)
- Unknown
- In another system, please specify: ___________ *(free text)*

**8.1b. Are the microbiological findings available in a structured and machine-readable format (e.g. FHIR®, CSV, HL7), so that software applications can identify, recognize and extract concrete data, incl. specific values and their internal structure?**

**Consider a Microsoft® Excel® or CSV-table with individual columns per parameter as a reference.** *(Please contact your hospital IT support, should you not be able to answer this question on your own. Consider using the free text field for further comments) (To be answered if answer to question 8 was “Electronically and on paper” or “[Only] electronically”) (Please select all that apply and provide a comment)*

- Yes, ___________ *(free text)*
- No, ___________ *(free text)*
- Unknown, ___________ *(free text)*

**8.2. Please specify, how microbiological results are available on paper.** *(To be answered if answer to question 8 was “Electronically and on paper” or “[Only] on paper”) (Please select all that apply)*

- Fax
- Patient file at the ward
- Other, [please specify:] ___________ *(free text)*

**9. Which strategies do you use [to conduct SSI surveillance]?** *(Please select the most appropriate answer per item)*

- Participation in clinical rounds on surgical wards
  - Never
  - Very rarely
  - Rarely
  - Occasionally
  - Frequently
  - Very frequently
  - Always
- Actively asking [for updates/information] from treating healthcare workers
  - Never
  - Very rarely
  - Rarely
  - Occasionally
  - Frequently
  - Very frequently
  - Always
- Review of microbiological findings
  - Never
  - Very rarely
  - Rarely
  - Occasionally
  - Frequently
  - Very frequently
  - Always
- Review of patients receiving antimicrobials
  - Never
  - Very rarely
  - Rarely
  - Occasionally
  - Frequently
  - Very frequently
  - Always
- Passively relying on [updates/information] from ward staff
  - Never
  - Very rarely
  - Rarely
  - Occasionally
  - Frequently
  - Very frequently
  - Always
- Other (please specify via follow up question)
  - Never
  - Very rarely
  - Rarely
  - Occasionally
  - Frequently
  - Very frequently
  - Always

**9.1. Please specify the “Other strategies” [for conducting SSI surveillance].** *(To be answered if answer regarding the item “Other” in question 9 was not “Never”) (Please provide an answer as free text)*

- ___________ *(free text)*

**10. Do you continue SSI surveillance for patients after hospital discharge (“post-discharge surveillance”)?** *(Please select one answer)*

- [Yes,] systematically for all patients (e.g. when re-admitted)
- [Yes,] information is collected if SSIs are detected conincidentally
- No
- Unknown

**10.1. How do you perform your post-discharge surveillance?** *(To be answered if answer to question 10 was “[Yes,]…”) (Please select all that apply)*

- Follow up phone calls to patients after defined time periods
- [Follow up] examination of patients after defined time periods
- Instruction of patients to inform the surgical department in case of SSI occurrence
- Exchange with ambulatory care providers
- Systematic review of reports and findings from the hospital emergency room and from hospital admissions (in case of re-admission [of operated patients])
- Other, [please specify:] ___________ *(free text)*

**10.2. Why do you not perform post-discharge surveillance?** *(To be answered if answer to question 10 was “No”) (Please select all that apply)*

- [Necessary] effort is too high
- Insufficient will of ambulatory care providers to cooperate
- Low relevance
- Other, [please specify:] ___________ *(free text)*

**Survey: Group B**

**1. Which hospital information system (HIS) do you use in your hospital?** *(Please select one answer)*

- SAP / i.s.h.med
- ORBIS
- NEXUS
- Cerner / Siemens Medico
- Unknown
- Other, [please specify:] ___________ *(free text)*

**2. Do you use an external infection prevention and control software?** *(Please select one answer)*

- Yes
- No
- Unknown

**2.1. Which one?** *(To be answered if answer to question 2 was “Yes”) (Please select one answer)*

- QS-MED (3M™)
- HyBASE® (epiNet AG)
- MetaIPSS (Cerner)
- MetaHMS (Cerner)
- MedReport (Dorner)
- ORBIS Infektionsmanagement (AGFA Healthcare)
- Other, [please specify:] ___________ *(free text)*

**3. Does your hospital provide IT support that assists you in your surveillance activities?** *(Please select one answer)*

- Yes
- No

**3.1. Does your hospital have IT support that could assist you in your surveillance activities?** *(To be answered if answer to question 3 was “No”) (Please select one answer)*

- Yes
- No
- Unknown

**3.1.1. Optional Question: Why do you not receive IT support for surveillance activities in your hospital?** *(To be answered if answer to question 3.1 was “No”) (Please provide an answer as free text)*

- ___________ *(free text)*

**4. Is the following information regarding operations available in your hospital information system (HIS)?** *(Please select all that apply)*

- Type of surgery (procedure code)
- Date of surgery
- Age (year of birth)
- Sex
- Date of hospital admission
- Date of hospital discharge
- ASA score
- Wound contamination class
- Duration of surgery
- Endoscopic (yes or no)
- Urgent procedure (yes or no)
- Revision surgery (yes or no)
- Implant (yes or no)
- Surgical site infection data
- Premature end of surveillance (due to reoperation or death)
- Unknown
- Other, [please specify:] ___________ *(free text)*

**5. Do you use an [external] data management software (e.g. Microsoft® Excel®) to conduct your surveillance?** *(Please select one answer)*

- Yes
- No
- Unknown

**5.1.** **Which one?** *(To be answered if answer to question 5 was “Yes”) (Please select one answer)*

- Microsoft® Excel®
- Other, [please specify:] ___________ *(free text)*

**6. Is it possible for you to export data from the hospital information system (HIS) (e.g. to Microsoft® Excel® or webKess)?** *(Please select one answer)*

- Yes, export to both [external] data management software (e.g. Microsoft® Excel®) and webKess
- Yes, export to [external] data management software (e.g. Microsoft® Excel®), but not to webKess
- Yes, export to webKess, but not to [external] data management software (e.g. Microsoft® Excel®)
- No export possible
- Unknown

**6.1. Which data can be exported?** *(To be answered if answer to question 6 was “Yes, …”) (Please select all that apply)*

- Type of surgery (procedure code)
- Date of surgery
- Age (year of birth)
- Sex
- Date of hospital admission
- Date of hospital discharge
- ASA score
- Wound contamination class
- Duration of surgery
- Endoscopic (yes or no)
- Urgent procedure (yes or no)
- Revision surgery (yes or no)
- Implant (yes or no)
- Surgical site infection data
- Premature end of surveillance (due to reoperation or death)
- Other, [please specify:] ___________ *(free text)*

**6.1.1. How is the allocation from surgical procedure code to KISS indicator procedure performed (e.g. procedure code 5-455.1 corresponds to KISS indicator procedure “COLO”)?** *(To be answered if “Type of surgery (procedure code)” was selected in question 6.1) (Please select one answer)*

- Automatically through the export from the hospital information system (HIS)
- Manually by [staff] (e.g. paper-based, via an [external] data management software (e.g. Microsoft® Excel®)
- Automatically [through] a direct import of the procedure code into webKess
- Unknown
- Other, [please specify:] ___________ *(free text)*

**6.2. Which [exportable parameters]/data must be manually edited before they can be entered or imported into webKess?** *(To be answered if answer to question 6 was “Yes, export to [external] data management software (e.g. Microsoft® Excel®), but not to webKess”) (Please select all that apply)*

- Allocation of procedure code to KISS indicator procedure (e.g. procedure code 5-455.1 corresponds to KISS indicator procedure “COLO”)
- Date of surgery
- Age (year of birth)
- Sex
- Date of hospital admission
- Date of hospital discharge
- ASA score
- Wound contamination class
- Duration of surgery
- Endoscopic (yes or no)
- Urgent procedure (yes or no)
- Revision surgery (yes or no)
- Implant (yes or no)
- Surgical site infection data
- Premature end of surveillance (due to reoperation or death)
- Unknown
- Other, [please specify:] ___________ *(free text)*

**7. Why has your department not used the webKess import functionality for [surveillance] data last year?** *(Please select all that apply)*

- Local IT deficits
- Lack of technical expertise
- Changes to the import specification
- Change of the hospital information system (HIS)
- Change of personnel
- Change in the way surveillance is conducted
- Lack of necessity
- Changes to the OP-KISS protocol
- Changes to the surgical procedure codes
- Other, [please specify:] ___________ *(free text)*

**8. In which form are microbiological results (e.g. wound swab, blood culture) necessary for OP-KISS surveillance available?** *(Please select one answer)*

- Electronically and on paper
- [Only] electronically
- [Only] on paper
- Not available at all

**8.1a. Please specify, how microbiological results are available electronically.** *(To be answered if answer to question 8 was “Electronically and on paper” or “[Only] electronically”) (Please select one answer)*

- In the hospital information system (HIS)
- Unknown
- In another system, please specify: ___________ *(free text)*

**8.1b. Are the microbiological findings available in a structured and machine-readable format (e.g. FHIR®, CSV, HL7), so that software applications can identify, recognize and extract concrete data, incl. specific values and their internal structure?**

**Consider a Microsoft® Excel® or CSV-table with individual columns per parameter as a reference.** *(Please contact your hospital IT support, should you not be able to answer this question on your own. Consider using the free text field for further comments) (To be answered if answer to question 8 was “Electronically and on paper” or “[Only] electronically”) (Please select all that apply and provide a comment)*

- Yes, ___________ *(free text)*
- No, ___________ *(free text)*
- Unknown, ___________ *(free text)*

**8.2. Please specify, how microbiological results are available on paper.** *(To be answered if answer to question 8 was “Electronically and on paper” or “[Only] on paper”) (Please select all that apply)*

- Fax
- Patient file at the ward
- Other, [please specify:] ___________ *(free text)*

**9. Which strategies do you use [to conduct SSI surveillance]?** *(Please select the most appropriate answer per item)*

- Participation in clinical rounds on surgical wards
  - Never
  - Very rarely
  - Rarely
  - Occasionally
  - Frequently
  - Very frequently
  - Always
- Actively asking [for updates/information] from treating healthcare workers
  - Never
  - Very rarely
  - Rarely
  - Occasionally
  - Frequently
  - Very frequently
  - Always
- Review of microbiological findings
  - Never
  - Very rarely
  - Rarely
  - Occasionally
  - Frequently
  - Very frequently
  - Always
- Review of patients receiving antimicrobials
  - Never
  - Very rarely
  - Rarely
  - Occasionally
  - Frequently
  - Very frequently
  - Always
- Passively relying on [updates/information] from ward staff
  - Never
  - Very rarely
  - Rarely
  - Occasionally
  - Frequently
  - Very frequently
  - Always
- Other (please specify via follow up question)
  - Never
  - Very rarely
  - Rarely
  - Occasionally
  - Frequently
  - Very frequently
  - Always

**9.1. Please specify the “Other strategies” [for conducting SSI surveillance].** *(To be answered if answer regarding the item “Other” in question 9 was not “Never”) (Please provide an answer as free text)*

- ___________ *(free text)*

**10. Do you continue SSI surveillance for patients after hospital discharge (“post-discharge surveillance”)?** *(Please select one answer)*

- [Yes,] systematically for all patients (e.g. when re-admitted)
- [Yes,] information is collected if SSIs are detected conincidentally
- No
- Unknown

**10.1. How do you perform your post-discharge surveillance?** *(To be answered if answer to question 10 was “[Yes,]…”) (Please select all that apply)*

- Follow up phone calls to patients after defined time periods
- [Follow up] examination of patients after defined time periods
- Instruction of patients to inform the surgical department in case of SSI occurrence
- Exchange with ambulatory care providers
- Systematic review of reports and findings from the hospital emergency room and from hospital admissions (in case of re-admission [of operated patients])
- Other, [please specify:] ___________ *(free text)*

**10.2. Why do you not perform post-discharge surveillance?** *(To be answered if answer to question 10 was “No”) (Please select all that apply)*

- [Necessary] effort is too high
- Insufficient will of ambulatory care providers to cooperate
- Low relevance
- Other, [please specify:] ___________ *(free text)*

**Survey: Group C**

**1. Which hospital information system (HIS) do you use in your hospital?** *(Please select one answer)*

- SAP / i.s.h.med
- ORBIS
- NEXUS
- Cerner / Siemens Medico
- Unknown
- Other, [please specify:] ___________ *(free text)*

**2. Do you use an external infection prevention and control software?** *(Please select one answer)*

- Yes
- No
- Unknown

**2.1. Which one?** *(To be answered if answer to question 2 was “Yes”) (Please select one answer)*

- QS-MED (3M™)
- HyBASE® (epiNet AG)
- MetaIPSS (Cerner)
- MetaHMS (Cerner)
- MedReport (Dorner)
- ORBIS Infektionsmanagement (AGFA Healthcare)
- Other, [please specify:] ___________ *(free text)*

**3. Does your hospital provide IT support that assists you in your surveillance activities?** *(Please select one answer)*

- Yes
- No

**3.1. Does your hospital have IT support that could assist you in your surveillance activities?** *(To be answered if answer to question 3 was “No”) (Please select one answer)*

- Yes
- No
- Unknown

**3.1.1. Optional Question: Why do you not receive IT support for surveillance activities in your hospital?** *(To be answered if answer to question 3.1 was “No”) (Please provide an answer as free text)*

- ___________ *(free text)*

**4. Is the following information regarding operations available in your hospital information system (HIS)?** *(Please select all that apply)*

- Type of surgery (procedure code)
- Date of surgery
- Age (year of birth)
- Sex
- Date of hospital admission
- Date of hospital discharge
- ASA score
- Wound contamination class
- Duration of surgery
- Endoscopic (yes or no)
- Urgent procedure (yes or no)
- Revision surgery (yes or no)
- Implant (yes or no)
- Surgical site infection data
- Premature end of surveillance (due to reoperation or death)
- Unknown
- Other, [please specify:] ___________ *(free text)*

**5. Do you use an [external] data management software (e.g. Microsoft® Excel®) to conduct your surveillance?** *(Please select one answer)*

- Yes
- No
- Unknown

**5.1.** **Which one?** *(To be answered if answer to question 5 was “Yes”) (Please select one answer)*

- Microsoft® Excel®
- Other, [please specify:] ___________ *(free text)*

**6. Is it possible for you to export data from the hospital information system (HIS) (e.g. to Microsoft® Excel® or webKess)?** *(Please select one answer)*

- Yes, export to both [external] data management software (e.g. Microsoft® Excel®) and webKess
- Yes, export to [external] data management software (e.g. Microsoft® Excel®), but not to webKess
- Yes, export to webKess, but not to [external] data management software (e.g. Microsoft® Excel®)
- No export possible
- Unknown

**6.1. Which data can be exported?** *(To be answered if answer to question 6 was “Yes, …”) (Please select all that apply)*

- Type of surgery (procedure code)
- Date of surgery
- Age (year of birth)
- Sex
- Date of hospital admission
- Date of hospital discharge
- ASA score
- Wound contamination class
- Duration of surgery
- Endoscopic (yes or no)
- Urgent procedure (yes or no)
- Revision surgery (yes or no)
- Implant (yes or no)
- Surgical site infection data
- Premature end of surveillance (due to reoperation or death)
- Other, [please specify:] ___________ *(free text)*

**6.1.1. How is the allocation from surgical procedure code to KISS indicator procedure performed (e.g. procedure code 5-455.1 corresponds to KISS indicator procedure “COLO”)?** *(To be answered if “Type of surgery (procedure code)” was selected in question 6.1) (Please select one answer)*

- Automatically through the export from the hospital information system (HIS)
- Manually by [staff] (e.g. paper-based, via an [external] data management software (e.g. Microsoft® Excel®)
- Automatically [through] a direct import of the procedure code into webKess
- Unknown
- Other, [please specify:] ___________ *(free text)*

**6.2. Which [exportable parameters]/data must be manually edited before they can be entered or imported into webKess?** *(To be answered if answer to question 6 was “Yes, export to [external] data management software (e.g. Microsoft® Excel®), but not to webKess”) (Please select all that apply)*

- Allocation of procedure code to KISS indicator procedure (e.g. procedure code 5-455.1 corresponds to KISS indicator procedure “COLO”)
- Date of surgery
- Age (year of birth)
- Sex
- Date of hospital admission
- Date of hospital discharge
- ASA score
- Wound contamination class
- Duration of surgery
- Endoscopic (yes or no)
- Urgent procedure (yes or no)
- Revision surgery (yes or no)
- Implant (yes or no)
- Surgical site infection data
- Premature end of surveillance (due to reoperation or death)
- Unknown
- Other, [please specify:] ___________ *(free text)*

**7.1. Why does your department use the webKess import functionality for [surveillance] data?** *(Please select all that apply)*

- Time saving
- Reduction of workload
- Utilization of preexisting internal hospital structures (e.g. use of internal software with OP-KISS export functionality)
- Directive from hospital management or head of department
- Quality management
- Other, [please specify:] ___________ *(free text)*

**7.1.1. Can you name the software that you use for the OP-KISS import?** *(To be answered if “Utilization of preexisting internal hospital structures (e.g. use of internal software with OP-KISS export functionality” was selected in question 7.1) (Please select one answer)*

- Yes
- No

**7.1.1.1. Which software do you use for the OP-KISS import?** *(To be answered if answer to question 7.1.1. was “Yes”) (Please provide an answer as free text)*

- ___________ *(free text)*

**7.2. Do you import [data on] surgical site infections on addition to [data on] surgeries [(i.e. denominator data)]?** *(Please select one answer)*

- Yes
- No
- Unknown

**7.2.1. Why do you not import** **[data on] surgical site infections?** *(To be answered if answer to question 7.2. was “No”) (Please select all that apply)*

- Technical prerequisites are not fulfilled locally
- Import specification is too complicated
- Reduction of workload is too low
- Error-proneness is too high
- Unknown
- Other, [please specify:] ___________ *(free text)*

**8. In which form are microbiological results (e.g. wound swab, blood culture) necessary for OP-KISS surveillance available?** *(Please select one answer)*

- Electronically and on paper
- [Only] electronically
- [Only] on paper
- Not available at all

**8.1a. Please specify, how microbiological results are available electronically.** *(To be answered if answer to question 8 was “Electronically and on paper” or “[Only] electronically”) (Please select one answer)*

- In the hospital information system (HIS)
- Unknown
- In another system, please specify: ___________ *(free text)*

**8.1b. Are the microbiological findings available in a structured and machine-readable format (e.g. FHIR®, CSV, HL7), so that software applications can identify, recognize and extract concrete data, incl. specific values and their internal structure?**

**Consider a Microsoft® Excel® or CSV-table with individual columns per parameter as a reference.** *(Please contact your hospital IT support, should you not be able to answer this question on your own. Consider using the free text field for further comments) (To be answered if answer to question 8 was “Electronically and on paper” or “[Only] electronically”) (Please select all that apply and provide a comment)*

- Yes, ___________ *(free text)*
- No, ___________ *(free text)*
- Unknown, ___________ *(free text)*

**8.2. Please specify, how microbiological results are available on paper.** *(To be answered if answer to question 8 was “Electronically and on paper” or “[Only] on paper”) (Please select all that apply)*

- Fax
- Patient file at the ward
- Other, [please specify:] ___________ *(free text)*

**9. Which strategies do you use [to conduct SSI surveillance]?** *(Please select the most appropriate answer per item)*

- Participation in clinical rounds on surgical wards
  - Never
  - Very rarely
  - Rarely
  - Occasionally
  - Frequently
  - Very frequently
  - Always
- Actively asking [for updates/information] from treating healthcare workers
  - Never
  - Very rarely
  - Rarely
  - Occasionally
  - Frequently
  - Very frequently
  - Always
- Review of microbiological findings
  - Never
  - Very rarely
  - Rarely
  - Occasionally
  - Frequently
  - Very frequently
  - Always
- Review of patients receiving antimicrobials
  - Never
  - Very rarely
  - Rarely
  - Occasionally
  - Frequently
  - Very frequently
  - Always
- Passively relying on [updates/information] from ward staff
  - Never
  - Very rarely
  - Rarely
  - Occasionally
  - Frequently
  - Very frequently
  - Always
- Other (please specify via follow up question)
  - Never
  - Very rarely
  - Rarely
  - Occasionally
  - Frequently
  - Very frequently
  - Always

**9.1. Please specify the “Other strategies” [for conducting SSI surveillance].** *(To be answered if answer regarding the item “Other” in question 9 was not “Never”) (Please provide an answer as free text)*

- ___________ *(free text)*

**10. Do you continue SSI surveillance for patients after hospital discharge (“post-discharge surveillance”)?** *(Please select one answer)*

- [Yes,] systematically for all patients (e.g. when re-admitted)
- [Yes,] information is collected if SSIs are detected conincidentally
- No
- Unknown

**10.1. How do you perform your post-discharge surveillance?** *(To be answered if answer to question 10 was “[Yes,]…”) (Please select all that apply)*

- Follow up phone calls to patients after defined time periods
- [Follow up] examination of patients after defined time periods
- Instruction of patients to inform the surgical department in case of SSI occurrence
- Exchange with ambulatory care providers
- Systematic review of reports and findings from the hospital emergency room and from hospital admissions (in case of re-admission [of operated patients])
- Other, [please specify:] ___________ *(free text)*

**10.2. Why do you not perform post-discharge surveillance?** *(To be answered if answer to question 10 was “No”) (Please select all that apply)*

- [Necessary] effort is too high
- Insufficient will of ambulatory care providers to cooperate
- Low relevance
- Other, [please specify:] ___________ *(free text)*
